# Impact of Student Interest Groups' Simulation Activities on the Awareness of Underrepresented Specialties and Knowledge of Related Pathologies in a School With Limited Residency Programs and Exposure

**DOI:** 10.7759/cureus.83196

**Published:** 2025-04-29

**Authors:** Ariana S Johnson, Michael T Hsieh, Sharon Toor, Brandon Molligoda, Simo Kraguljac, Rees Ridout, David M Harris

**Affiliations:** 1 Medical Education, University of Central Florida College of Medicine, Orlando, USA

**Keywords:** medical education, medical student, otolaryngology, simulation, specialty selection

## Abstract

Introduction: Medical students who attend home institutions without specialty programs, like otolaryngology (ENT) and anesthesiology, have less awareness of the specialty. The purpose of this study was to determine if simulation activities developed by student interest groups in medical specialties can increase awareness and knowledge of these specialties.

Methods: First-, second- and third-year medical students participated in two simulations. The first simulation session was a collaboration between the ENT Interest Group at the University of Central Florida College of Medicine (UCF COM) and Stryker (Kalamazoo, Michigan, USA), a medical equipment vendor that supplied the surgical equipment. The hands-on simulation activities included a variety of ENT equipment. The second simulation session was a collaboration between the ENT Interest Group, Neurology Interest Group and Anesthesiology Interest Group with residents and physicians from affiliated institutions. Students participated in hands-on activities that included simulators and working with physicians on intubation, using an otoscope and reading head and neck medical images. In both, participants completed a Likert-scale questionnaire prior to and immediately following the hands-on simulation activity. Additionally, participants responded to one open-ended question. IBM SPSS Statistics, version 28.0 (IBM Corp., Armonk, USA) and paired t-test were used for data analysis.

Results: Thirty-six medical students completed the first simulation and survey. Interest in pursuing ENT rose from 2.75 to 3.25 (p<0.001), awareness of ENT instruments increased from 1.58 to 3.28 (p<0.001), and confidence in using these instruments increased from 1.31 to 2.89 (p<0.001). Knowledge of head and neck anatomy and ENT diseases also improved, with scores rising from 2.17 to 2.86 (p<0.001) and 2.17 to 2.92 (p<0.001), respectively. Students who reported increased interest in ENT attributed it primarily to the knowledge gained and the hands-on nature of the simulation. Twenty medical students completed the second simulation and survey. Interest increased significantly in neurology (1.75 to 2.75, p<0.05); however, medical knowledge and procedural knowledge were significantly increased in all three specialties of ENT, neurology and anesthesiology.

Conclusion: This study indicates that simulation-based events can substantially enhance both interest and awareness in ENT and anesthesiology, particularly for medical students with limited exposure to the specialty. By providing immersive, hands-on experiences, these simulations offer medical students valuable insights into specialties that are typically unavailable until clinical years.

## Introduction

It is well established that students receive insufficient exposure to otolaryngology (ENT) during their undergraduate medical education [1-4]. This educational gap is significant as ENT complaints account for 10% of new adult consultations and 50% of new pediatric consultations in general practice [5,6]. This issue is further compounded by recent medical school graduates reporting low comfort levels in managing ENT complaints [7]. Additionally, over 90% of medical students reported minimal classroom and clinical exposure to ENT during their undergraduate education [8].

ENT is regarded as one of the most competitive specialties. However, there were fewer applicants than the number of available positions in 2017 [9]. According to the 2016 Residency Matching Program data, the average Step 1 score was 248, much higher than the mean, which also most likely led to decreased numbers of students applying to the specialty [9]. At the same time, lower applicant numbers may be a result of the specialty’s increased competitiveness and a lack of awareness about ENT amongst medical students. In response, studies were conducted to investigate this momentary decrease, as well as the factors that influence medical students to choose ENT [10]. Results showed that medical schools with a home ENT program were more than twice as likely to have graduates enter ENT compared to those without home programs [10]. There was a positive correlation found between the presence of an ENT student interest group and an increase in the number of graduates entering this specialty [10]. This is critical for newer medical schools without an established ENT residency program as student-led groups can help cultivate interest in this specialty.

Additionally, multiple studies have been conducted to understand why students choose ENT as their specialty. One of the most significant factors was the variety of surgical procedures offered [11]. A systematic review found that work-life balance and positive role models were key contributors, while lack of exposure was a barrier [12]. Therefore, it is essential to develop creative approaches to increase medical students’ exposure to the ENT specialty.

Anesthesiology is another specialty that undergraduate medical students typically have limited exposure to early in medical education. Most medical school have retained the two-phase curriculum consisting of foundational sciences followed by clinical clerkships. The often-utilized systems-based structure does not allow for anesthesiology training. Additionally, many schools may not have the faculty to teach in the medical school program due to lack of residency programs and opportunities within the curriculum [13]. A study on Canadian medical schools showed that anesthesiology had approximately two hours of didactic time [14].

It takes time for newer medical schools to create residency programs, thus allowing less exposure for their students to some specialties at their home institution. The University of Central Florida College of Medicine (UCF COM) does not have a residency program in ENT or anesthesiology, and one residency program in neurology. Therefore, the student interest groups combined efforts to expose medical students to these specialties to improve exposure. The purpose of this study was to determine if simulation activities developed by student interest groups in medical specialties can increase awareness and knowledge of these specialties. The first simulation session was a collaboration between the ENT Interest Group at the UCF COM and Stryker (Kalamazoo, Michigan), a medical equipment vendor that supplied the surgical equipment. The second simulation session was a collaboration between the ENT Interest Group, Neurology Interest Group and Anesthesiology Interest Group with residents and physicians from affiliated institutions.

This article was previously presented as a podium presentation at the Triological Society Combined Sections Meeting, January 23-25, 2025, in Orlando, Florida.

## Materials and methods

Study sample

First-, second- and third-year medical students were recruited for the two simulation sessions via email and through social media. Fourth-year medical students (M4s) were excluded for recruitment since these simulations occurred in the Spring and M4s were prepared for the residency match. The University of Central Florida College of Medicine has about 120 medical students per year. There are no residency programs in ENT, but there is one in both Neurology and Anesthesiology.

Both simulation sessions were limited by room capacity and the opportunity for hands-on activities, thus creating a first-come, first served scenario that should be considered in relation to results.

Simulation One

The participant limit was set at 36 students based on room capacity and to ensure everyone had the opportunity for hands-on experience. The first 36 students who registered online were given the opportunity to attend the event. Among those in attendance, 25 were first-year students, 10 were second-year students, and 1 was a third-year student. Any medical student who was a first-, second- or third-year student could participate. There were no exclusion criteria.

Simulation Two

The participant limit was set at 20 students based on room capacity. Similar to Simulation One, the first 20 students who registered online were given the opportunity to attend the event. There were 15 first-year medical students and 5 second-year medical students. Any medical student who was a first-, second- or third-year student was invited to participate. There were no exclusion criteria.

The study was determined to be Exempt from Regulation by the University of Central Florida Institutional Review Board (STUDY00006460).

Study design and setting

The study used a pre- and post-test study design for the two simulation activities. Participants accessed the survey tests using a QR code located at the front of the room. The pre-test was provided at the beginning of each session before any instructional activity or hands-on activities were done. The post-test was completed at the end of each session.

Simulation One

The research took place in a classroom at the University of Central Florida Health Sciences Building that had adequate space for a didactic portion as well as space for simulation equipment. Students completed a pre-test consisting of five Likert-scale questions immediately before the session. The post-test was administered after the completion of the simulation and consisted of the same five Likert-scale questions along with one additional open-ended question. The questions addressed topics such as interest in pursuing ENT, knowledge of head and neck anatomy and disease, awareness of ENT surgical tools and confidence in utilizing them.

Simulation Two

The research was conducted at the Clinical Skills and Simulation Center at the University of Central Florida Health Sciences Building. This Center has a small section for didactic training as well as patient rooms utilized for clinical skills development. Students completed a pre-test consisting of four Likert-scale questions immediately before the hands-on simulation activity. The post-test consisted of the same four questions and was given at the conclusion of the simulation session.

Description of simulation sessions

Simulation One

**Figure 1:**
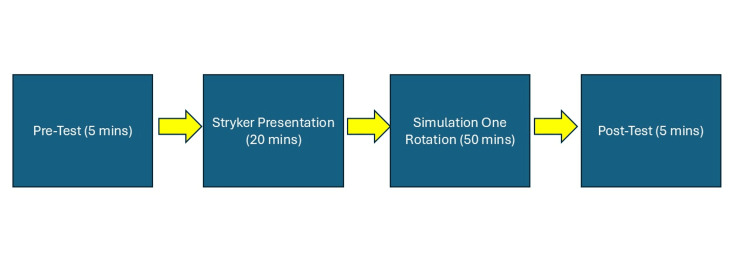
Schematic of Simulation One

The ENT Interest Group partnered with Stryker, a medical equipment company, to deliver a 20-minute presentation covering head and neck anatomy and the pathology of four common ENT conditions: chronic sinusitis, chronic rhinitis, nasal airway obstruction, and eustachian tube dysfunction (ETD). The Stryker representatives provided an overview of the ENT surgical tools that would be used by participants to simulate the treatment of these conditions. Participants spent the next 50 minutes rotating through the stations with the guidance of the representatives. The stations included XprESS Sinus Dilation utilizing the Scopis Navigation System and FocESS camera for chronic sinusitis treatment, ClariFix Cryotherapy for chronic rhinitis treatment, LATERA Nasal Implant for treatment of nasal airway obstruction and Eustachian Tube Dilation for ETD management. The schematic for Simulation One is shown in Figure 1.

Simulation Two

**Figure 2:**
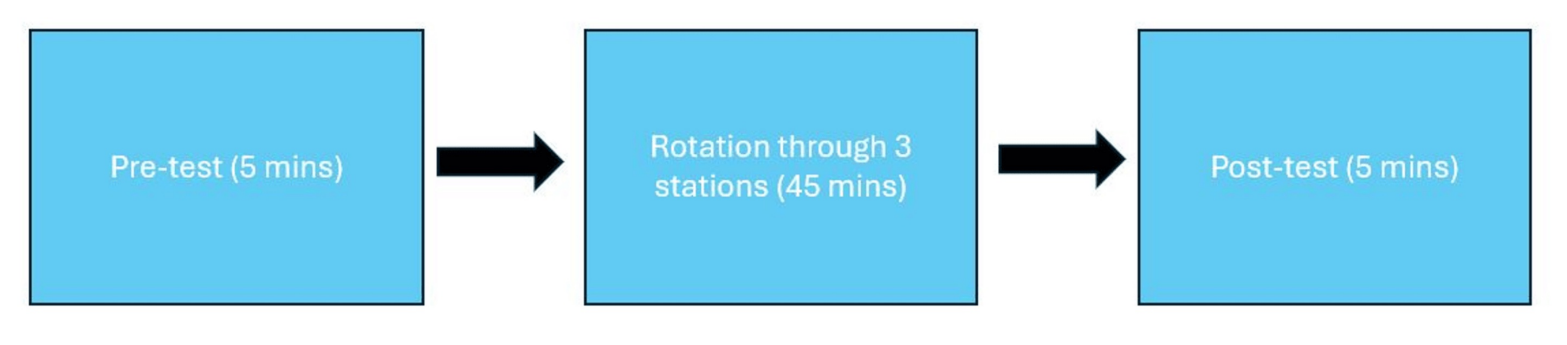
Schematic for Simulation Two

The ENT interest group partnered with the Neurology and Anesthesiology Interest Groups and their respective resident physicians to design the activities for Simulation Two. After students did the pre-test at the beginning of the activity, they then rotated through a series of simulation activities with physicians. The three rotations were ear pathologies with otoscopes, neurology imaging, and an intubation workshop for 45 minutes. There was a physician to help students in each session work with the simulator or images. This was followed by the post-test. The schematic for Simulation Two is shown in Figure 2.

Data analysis

A paired samples t-test was used to compare the change in scores before and after the simulation sessions. IBM SPSS Statistics, version 28.0 (IBM Corp., Armonk, USA) was used for data analysis with p<0.05 considered statistically significant. For the narrative data, representative samples were selected by the authors.

## Results

Simulation One

Interest in Otolaryngology

**Figure 3:**
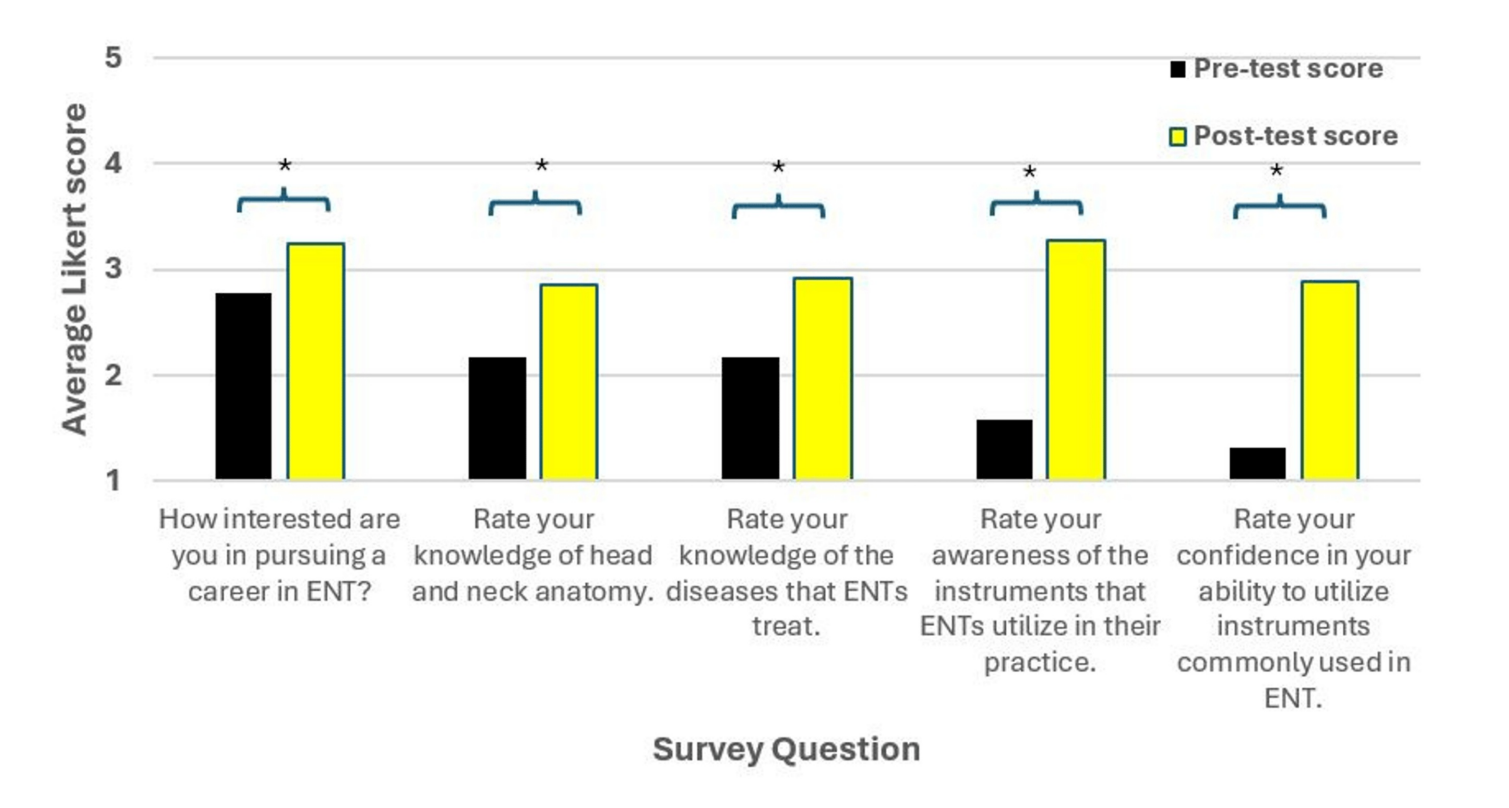
Survey questions for Simulation One This figure shows the pre- and post-test scores for Simulation One from the survey. There was a significant difference between the pre- and post-test scores for all questions denoted by the asterisk (*p<0.05). The two knowledge-based questions used the following choices: 1 = no knowledge, 2 = limited knowledge, 3 = somewhat knowledgeable, 4 = very knowledgeable, and 5 = extremely knowledgeable. The other questions used similar terminology with 1 = no interest/confidence/awareness, 2 = limited interested/confident/aware, 3 = somewhat interested/confident/aware, 4 = very interested/confident/aware or 5 = extremely interested/confident/aware.

The first question assessed students’ interest in ENT prior to participation in the simulation. Figure 3 shows a statistically significant increase in specialty interest, rising from 2.78 to 3.25 (n=36, p<0.001; 95% CI, -0.644, -0.301). One of the three students who originally reported no interest became slightly interested following the session. These findings suggest that the simulation successfully increased student interest in ENT.

Knowledge of Head and Neck Anatomy and Disease

The next set of questions were provided to determine whether participants’ knowledge of relevant head and neck anatomy, as well as the diseases treated by ENT physicians, had increased. Notably, most of the participants were first-year medical students who prior to the session had not yet engaged in head and neck anatomy in the lab. The ratings for knowledge of head and neck anatomy significantly increased from 2.17 to 2.86 (n=36, p<0.001; 95% CI, -0.906, -0.483), while the ratings for knowledge of ENT diseases significantly increased from 2.17 to 2.92 (n=36, p<0.001; 95% CI, -0.970, -0.530). These data suggest that the simulation increased participants’ knowledge of head and neck anatomy and ENT diseases.

Awareness of ENT Surgical Instruments and Confidence in Their Use

The next set of questions aimed to gauge students’ awareness of ENT surgical instruments as well as their confidence in utilizing the instruments. The following scale was used for awareness of the ENT instruments: 1 = no awareness, 2 = limited awareness, 3 = somewhat aware, 4 = very aware, and 5 = extremely aware. Awareness of instruments significantly increased from 1.58 to 3.28 (n=36, p<0.001; 95% CI, -1.948, -1.441). Confidence level increased significantly from 1.31 to 2.89 (n=36, p<0.001; 95% CI, -1.831, -1.336). These data suggest that the simulation enhanced awareness of the instruments used by ENT physicians and increased student confidence in their use.

Open-Ended Question Results

Following the session, the participants were asked the following open-ended question: “If you rated your interest in pursuing ENT higher than before, then what about this event piqued your interest?”. In total, 31 of the 36 students provided a written response. Table 1 highlights representative responses reflecting student experience. These responses emphasized the hands-on use of the equipment and procedure simulation as critical factors in capturing participants’ interest in ENT.

**Table 1:** Responses to the open-ended question, "If you rated your interest in pursuing ENT higher than before, then what about this event piqued your interest?"

Example	Response
1	“Seeing the tools used is pretty cool and seeing how a simple procedure can solve a problem that a patient may had been having for a long time!”
2	“It was really interesting to learn about the tools and how they can help patients. I also loved trying them out.”
3	“The tools and the innovation definitely peaked my interest in ENT more than before.”
4	“The procedural nature, and the anatomy that I can interact with. Also, the flexibility in careers.”
5	“It definitely did, I loved the hands-on and interactive experience, it made it so unique and in my opinion more valuable than a typical talk.”
6	“I am more comfortable with the surgery aspect of ENT and can envision myself doing those procedures.”

Simulation Two

In Simulation Two, the ENT student interest group partnered with interest groups in neurology and anesthesiology to develop a series of simulation activities focusing on these specialties.

Specialty Interest

**Figure 4:**
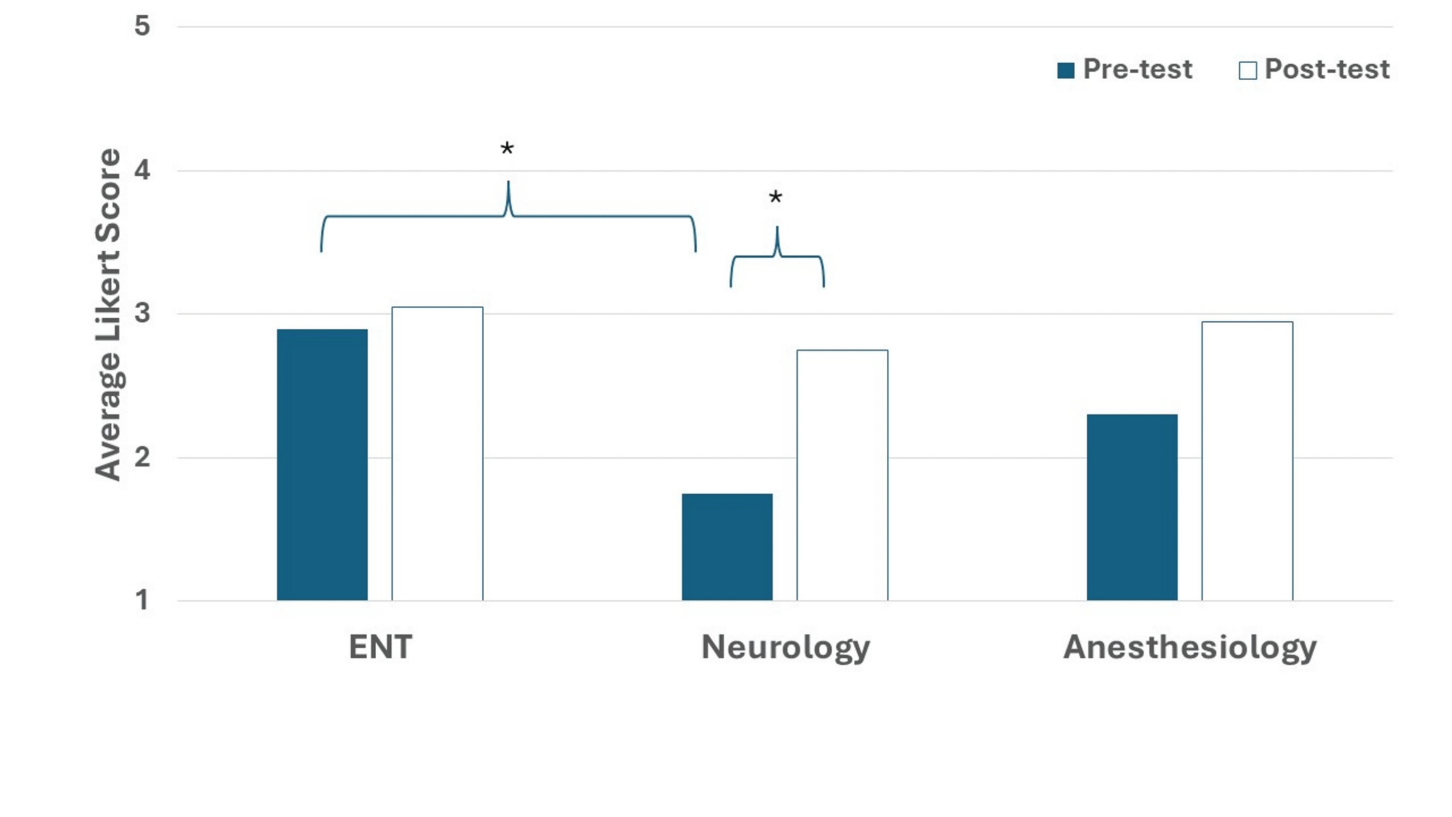
Specialty interest for Simulation Two The prompt in the survey was, "How interested are you in pursuing otolaryngology, neurology or anesthesiology as a specialty?" *p<0.05

In Simulation Two, participants were asked to rate their interest in the specialties of ENT, neurology and anesthesiology before and after the simulation encounters. Fifteen first-year and five second-year medical students fully completed both the pre- and post-test surveys. Participants were assessed for their interest in specialties including ENT, neurology, and anesthesiology; data are shown in Figure 4. Prior to our simulation, participants were more interested in ENT (2.90 ± 1.21) compared to anesthesiology (2.30 ± 1.03, p=0.10) and significantly compared to neurology (1.75 ± 0.79, p<0.01). After the simulation, students showed a rise in interest in ENT (3.05 ± 1.28, change of 0.15), anesthesiology (2.95 ± 1.23, change of 0.65) and neurology (2.75 ± 1.07, p<0.01, change of 1.00), although it was only significant for neurology. This suggests that the simulation may have had more of an effect on neurology interest.

Medical and Procedural Knowledge

Participants were asked to rate statements regarding medical knowledge during Simulation Two. These statements were related to head and neck anatomy and pathology. The first four statements in Table 2 show that there was a significant increase in knowledge scores related to the three specialty disciplines. The last three statements were related to procedural knowledge that students learned during the simulation activity. There was a significant increase in confidence for each of the three procedures as can be seen in Table 2.

**Table 2:** Perceptions of medical and procedural knowledge Students rated themselves on a scale of knowledgeable or confidence, where 1 = not at all knowledgeable/confident, 2 = slightly knowledgeable/confident, 3 = moderately knowledgeable/confident, 4 = very knowledgeable/confident, 5 = extremely knowledgeable/confident. The asterisk represents a significant change, p<0.05.

Statement	Pre-test average (SD)	Post-test average (SD)
I am knowledgeable about how head and neck pathologies may present differently in children vs. adults.	2.00 (0.7)	3.15 (0.9)*
I am confident identifying key anatomical structures of the head and neck that are linked to pathology.	2.55 (0.8)	3.70 (0.7)*
I am confident with distinguishing head and neck pathologies based on imaging.	2.10 (0.8)	3.55 (0.6)*
I am confident describing how neurological function can be affected by head and neck surgeries.	2.50 (0.7)	3.30 (0.9)*
I am confident in reading head and neck films.	1.95 (0.6)	3.30 (0.8)*
I am confident in intubating patients.	1.95 (0.8)	3.65 (0.7)*
I am confident using an otoscope properly to identify ear structures.	2.9 (0.7)	3.75 (0.6)*

## Discussion

The findings from the current study show that student interest groups can use simulation activities outside of the formal curriculum to increase awareness and knowledge related to specialties, such as ENT, which are underrepresented in undergraduate medical education. Narrative responses showed the positive impact that hands-on experience with surgical equipment had on increasing student interest in pursuing ENT. The data from this study also suggest that hands-on activities facilitate interest in neurology and anesthesiology in early learners as well.

This study aligns with the earlier research on student-run free clinics to introduce medical students to the field of ENT [15]. That study reported increased knowledge of ENT and improved confidence in performing head and neck exams following lecture and skills practice [15]. A major difference between the current study and prior research is the involvement of ENT residents in running the free clinics, while UCF COM does not have ENT home residents. Therefore, our findings provide an alternative perspective on the experience of students who lack direct access to ENT residents for guidance and mentorship. Our data suggest that the model utilized by the student interest group in Simulation One could serve as a model to address disparities in medical education in the future.

Narrative responses from our study highlighted the value of hands-on experience with surgical instruments and simulation of procedures as strengths of the event. Other surgical simulations, such as 3D myringotomy and tube placement, have previously been used to introduce medical students to ENT [16]. While the myringotomy study emphasized improving student knowledge of tube placement, this study shares the common goal of introducing students to the specialty via surgical simulation [16]. Key similarities between our study and the aforementioned one is the larger portion of first-year medical students with limited clinical experience. One key difference between our study and the myringotomy study was the availability of otolaryngologists to guide the students during the simulation activity. Despite the lack of a residency program and access to laryngologists, our participants' knowledge improved. Due to the positive feedback of students and the results of this simulation, more interactive activities should be offered to expose students to ENT and other underrepresented specialties, particularly in medical schools that lack these residency programs.

The current study provides a novel and feasible method to introduce students to the field of ENT. A recent study by Gawel et al. introduced a flexible laryngoscope workshop for first- and second-year medical students [17]. In that study, they found that interest and familiarity with ENT increased after their workshop that consisted of a 10-minute didactic session followed by a 20-minute hands-on activity with resident physicians using the laryngoscope [17]. Our results were similar in that we observed significant improvements in interest in the specialty as well as knowledge about pathologies. Their group also partnered with a vendor, Ambu (Columbia, Maryland) to aid. Taken together, the studies suggest that student interest groups partnering with vendors of equipment may offer a reasonable way to increase exposure to the field.

The current study also provides data to suggest that exposure to anesthesiology through a hands-on activity is beneficial for student interest and knowledge about it. Baylor University Medical Center has developed a summer preceptorship covering anesthesiology due to its increasing demand [18]. Their program offers hands-on experience with intubation, similar to our study, and also other techniques such as cannulation and ultrasound techniques. All students were able to do endotracheal intubation at the end of their four-week program. Although a full program is most likely not feasible at our institution, the student interest group of anesthesiology could mimic this program and its activities to determine if similar increases can be attained in a modified setup.

There are several limitations to the current study. One limitation is that students self-selected for participation in these events. Although most students did not initially express a strong interest in ENT, neurology or anesthesiology, we cannot predict if the same results would apply to the total student population. The small size of the study is a limitation although it could potentially be scaled higher if more interest is noted. However, the specialty interest groups were restricted by available space, and increasing the number of participants would have negatively affected the quality of hands-on experience. Future studies could include randomized recruitment methods or increase the capacity to include more students.

Another limitation is that the first event was hosted at a medical school without an ENT residency program with limited access to ENT residents and faculty. It is unknown whether the results of the simulation experiences would be similar if led by residents as opposed to medical equipment representatives. Future studies could be developed to look at the potential differences between physician-led or vendor-led simulation sessions to determine if there are any changes in educational effectiveness.

Also, Likert-scale questions were used to measure the tested variables. Future studies could utilize other methods such as focus groups, interviews, or tests other than perception to measure intended outcomes. Additionally, studies at later time points could assess the long-term effects of these types of interventions. However, the current study showed that the development of these sessions is feasible.

## Conclusions

Our study findings suggest that collaborations between student interest groups and medical equipment companies can provide simulation-based events to generate interest in specialties with limited exposure due to lack or low numbers of residency programs. The important components necessary for simulation events include clinically relevant knowledge directed towards specialties and hands-on activities. They also provide valuable opportunities to illustrate how anatomy can be applied to real-life scenarios.
